# Nitrogen Source and External Medium pH Interaction Differentially Affects Root and Shoot Metabolism in Arabidopsis

**DOI:** 10.3389/fpls.2016.00029

**Published:** 2016-02-01

**Authors:** Asier Sarasketa, M. Begoña González-Moro, Carmen González-Murua, Daniel Marino

**Affiliations:** ^1^Department of Plant Biology and Ecology, University of the Basque Country (UPV/EHU)Bilbao, Spain; ^2^Ikerbasque, Basque Foundation for ScienceBilbao, Spain

**Keywords:** ammonium, *Arabidopsis thaliana*, glutamate dehydrogenase, glutamine synthetase, nitrate, nitrogen metabolism, pH, TCA cycle

## Abstract

Ammonium nutrition often represents an important growth-limiting stress in plants. Some of the symptoms that plants present under ammonium nutrition have been associated with pH deregulation, in fact external medium pH control is known to improve plants ammonium tolerance. However, the way plant cell metabolism adjusts to these changes is not completely understood. Thus, in this work we focused on how *Arabidopsis thaliana* shoot and root respond to different nutritional regimes by varying the nitrogen source (${\text{NO}}_{3}^{-}$ and ${\text{NH}}_{4}^{+}$), concentration (2 and 10 mM) and pH of the external medium (5.7 and 6.7) to gain a deeper understanding of cell metabolic adaptation upon altering these environmental factors. The results obtained evidence changes in the response of ammonium assimilation machinery and of the anaplerotic enzymes associated to Tricarboxylic Acids (TCA) cycle in function of the plant organ, the nitrogen source and the degree of ammonium stress. A greater stress severity at pH 5.7 was related to ${\text{NH}}_{4}^{+}$ accumulation; this could not be circumvented in spite of the stimulation of glutamine synthetase, glutamate dehydrogenase, and TCA cycle anaplerotic enzymes. Moreover, this study suggests specific functions for different *gln* and *gdh* isoforms based on the nutritional regime. Overall, ${\text{NH}}_{4}^{+}$ accumulation triggering ammonium stress appears to bear no relation to nitrogen assimilation impairment.

## Introduction

Nitrate (${\text{NO}}_{3}^{-}$) and ammonium (${\text{NH}}_{4}^{+}$) comprise the main inorganic forms of nitrogen (N) absorbed by plants. The preference for either ${\text{NO}}_{3}^{-}$ or ${\text{NH}}_{4}^{+}$ as the N source is an important ecological determinant which affects plant diversity; while this aspect has not yet been precisely defined, it is however known to depend on environmental features such as soil properties (including pH), plant physiology and genetic background (van den Berg et al., 2005). Regardless of the N source, nitrogen is only incorporated into biomolecules as ${\text{NH}}_{4}^{+}$; however, paradoxically, an elevated abundance of this cation is toxic for plants, although the toxicity threshold greatly depends on ammonium concentration (Li et al., 2014). Symptoms experienced by plants when facing ammonium stress include chlorosis, ionic imbalance, reduced photosynthetic activity, changes in ${\text{NH}}_{4}^{+}$, amino acids, organic acids, and carbohydrates pool or pH deregulation.

Soil pH fluctuates widely between natural and agricultural soils and represents an important feature that may limit N availability and the plant's capacity to absorb essential nutrients (Marschner, 2012). Moreover, pH alterations may have an influence on cellular expansion (Cosgrove, 1999) and water conductance in roots (Kamaluddin and Zwiazek, 2004), besides other phenomena. Furthermore, H^+^s also play a role as second messengers in cell signaling cascades and so internal pH control is essential for the fine tuning of cells functioning (Felle, 2001). High ammonium content is common in acidic soils and the connection between ammonium stress and pH alteration has been known from a long time (Chaillou et al., 1991; Gerendás and Ratcliffe, 2000). Indeed ammonium-tolerant plants can sometimes also tolerate acidic conditions and controlling external medium pH has been shown to mitigate ammonium toxicity (Li et al., 2014).

${\text{NH}}_{4}^{+}$ uptake is known to induce acidification of the rhizosphere/apoplast, whereas ${\text{NO}}_{3}^{-}$ uptake promotes external alkalinization. Further to this it has been suggested that ${\text{NH}}_{4}^{+}$ uptake causes cytosolic alkalinization, while ${\text{NO}}_{3}^{-}$ uptake provokes cytosolic acidification (Marschner, 2012). However, this potential cytosolic alteration associated to N uptake is transient because when uptake and assimilation are considered as a whole process both nitrate and ammonium nutritions tend to alkalinize cell cytosol (Britto and Kronzucker, 2005). Indeed, although intracellular pH values are sensitive to external pH values, cytosolic pH is extremely stable thanks to the fine tuning of cell metabolism. This is evidenced by several studies which observed that external pH changes over a range of pH 4-10 had very little impact on internal cytoplasmic pH (Hartung and Ratcliffe, 2002; Gerendas and Ratcliffe, 2013). A further example is the work of Hachiya et al. (2012) who, by the use of *A. thaliana* plants expressing a cytosolic fluorescent pH sensor, observed that although apoplast pH decreased upon ammonium stress, cytosolic pH remained stable. Indeed, cell metabolic adjustment in response to changes in soil medium parameters, such as N source and availability, is crucial for plants in order to maintain their growth rates and fitness.

${\text{NO}}_{3}^{-}$ is reduced to ${\text{NH}}_{4}^{+}$ by nitrate and nitrite reductases; subsequently ammonium is mainly incorporated into amino acids via the glutamine synthetase/glutamate synthase (GS/GOGAT) cycle in which both nutrition pathways (${\text{NO}}_{3}^{-}$ and ${\text{NH}}_{4}^{+}$) converge. Nevertheless, it has been proposed that under some circumstances NADH-glutamate dehydrogenase (GDH), enzyme that catalyzes the reversible deamination of glutamate to 2-oxoglutarate (2-OG) could also collaborate in ${\text{NH}}_{4}^{+}$ assimilation (Ferraro et al., 2015). Nitrogen assimilation is intertwined with the respiratory metabolism; and it is known that the Tricarboxilic Acids (TCA) cycle and its associated anaplerotic enzymes play a central role (re)generating 2-OG for ${\text{NH}}_{4}^{+}$ assimilation. Indeed, several studies have highlighted the importance of a suitable carbon supply to alleviate ${\text{NH}}_{4}^{+}$ toxicity by controlling/modulating environmental conditions in order to favor carbon assimilation (Roosta and Schjoerring, 2008; Setién et al., 2013; Vega-Mas et al., 2015).

In general, external medium pH control (buffering or alkalinization) has been shown to mitigate some of the symptoms associated with ammonium stress, but how *Arabidopsis thaliana* ammonium assimilation machinery adapts to those pH changes is scarcely known. Thus, the aim of this work was to study the relationship between plants performance and cell metabolic adjustment under different nutritional regimes; combining nitrogen source (ammonium or nitrate), concentration (2 or 10 mM) and external medium pH (5.7 or 6.7). We focused on GS and GDH enzyme response together with TCA anaplerotic enzymes in both shoot and root. The overall results reveal that external medium pH strongly determines Arabidopsis response in function of the nitrogen source. Moreover, the pH-dependent differential ${\text{NH}}_{4}^{+}$ accumulation appears to set ammonium stress degree.

## Materials and methods

### Experimental procedure and growth conditions

*A. thaliana* Col-0 seeds were surface sterilized and sown in 0.6% agar Petri dishes with a modified MS solution (2.25 mM CaCl_2_, 1.25 mM KH_2_PO_4_, 0.75 mM MgSO_4_, 5 mM KCl, 0.085 mM Na_2_EDTA, 5 μM KI, 0.1 μM CuSO_4_, 100 μM MnSO_4_, 100 μM H_3_BO_3_, 0.1 μM CoCl_2_, 100 μM FeSO_4_, 30 μM ZnSO_4_, and 0.1 μM Na_2_MoO_4_; 20.5 mM MES, pH 5.7) containing 1 mM of NH_4_NO_3_ and 0.5% sucrose. Plates were kept during 4 days in the dark at 4°C and then moved into a controlled conditions phytotron: 14 h, 200 μmol m^−2^ s^−1^ light intensity, 60% RH and 22°C day conditions and 10 h, 70% RH, and 18°C night conditions.

Nine day-old seedlings were transferred to 24-well plates containing 1 ml of nutrient solution (1 plant/well). Eight different treatments were assayed, all of them with the same MS-solution used for germination but varying pH (5.7 or 6.7), N-source (${\text{NH}}_{4}^{+}$ or ${\text{NO}}_{3}^{-}$) and N concentration (2 and 10 mM). ${\text{NH}}_{4}^{+}$ was provided as (NH_4_)_2_SO_4_ and nitrate as Ca(NO_3_)_2_. To properly compare different N nutritions, ${\text{NH}}_{4}^{+}$-fed plants were supplemented with 1 or 5 mM CaSO_4_ to compensate the Ca^2+^ supplied together with the ${\text{NO}}_{3}^{-}$. Two pH regimes were selected with the objective to study a moderate pH change in the growth medium. Standard MS pH is 5.7-5.9, so we chose 5.7 as low pH and 6.7 as high in order to maintain the acidic nature of apoplastic pH (Felle, 2001) remaining within the range of the buffering capacity of MES (5.5–6.7).

Plates were kept under continuous shaking (120 rpm) during 12 days. The nutrient solution was renewed in days 5 and 9 and the evolution of the pH of the external medium monitored (Supplementary Figure 1). Sterility was maintained until harvest. Six independent experiments were performed. In each experiment six 24-well plates were analyzed, each plate containing three plants per treatment. When harvesting, shoots and roots were dried with paper towels, biomass recorded and immediately frozen in liquid nitrogen and stored at −80°C. Biomass was determined as the mean value of three plants grown in the same plate as one biological replicate.

### Ammonium and total amino acids determination

Tissue accumulation of ammonium and total amino acid content were determined as described in Sarasketa et al. (2014) following ninhydrin method for free amino acids determination and phenol hypochlorite assay for ammonium quantification. Glutamine was used as standard for the calibration curve for total amino acid content determination.

### Protein extraction and quantification

Leaves and roots were homogenized using a mortar and pestle with 20 μL of extraction buffer per mg of FW [10 mM MgCl_2_, 1 mM EDTA,1 mM EGTA, 10 mM dithiothreitol (DTT), 0.1% Triton X-100, 10% glycerol, 0.05% bovine serum albumin (BSA), 0.5% polyvinylpolypyrrolidone (PVPP), 50 mM HEPES pH 7.5] in the presence of a cocktail of proteases inhibitors [1 mM phenylmethylsulfonyl fluoride (PMSF), 1 mM ε-aminocaproic acid, 10 μM leupeptin]. Homogenates were then centrifuged at 4,000 *g* for 30 min at 4°C and the supernatants recovered. Soluble protein content was determined by a dye binding protein assay (Bio-Rad Bradford Protein assay) with BSA as standard for the calibration curve.

### Enzyme activities

For all the enzymes determined, except for glutamine synthetase (GS), 20 μL of protein extraction supernatants were incubated with 280 μL of reaction buffer in 96-well microplates and the evolution of NAD(P)H was spectrophotometically monitored at 340 nm during 20 min at 30°C. The reaction buffers were for NAD(H)-GDH: 100 mM Tris-HCl (pH 8), 1 mM CaCl_2_, 13 mM 2-oxoglutarate, 50 mM (NH_4_)_2_SO_4_, and 0.25 mM NADH; for NADH-dependent glutamate synthase (GOGAT): 100 mM Tricine-KOH (pH 8.6), 0.2 mM NADH, 10 mM DTT, 1 mM 2-oxoglutarate, 3 mM glutamine; for phosphoenolpyruvate carboxylase (PEPC): 100 mM Tricine-KOH (pH 8), 5 mM MgCl_2_, 5 mM NaF, 0.25 mM NADH, 6.4 U of malate dehydrogenase/mL, 2 mM NaHCO_3_ and 3 mM phosphoenolpyruvate; for MDH: 100 mM HEPES-KOH pH (7.5), 5 mM MgSO_4_, 0.2 mM NADH, 2 mM oxaloacetate; for NAD-dependent malic enzyme (NAD-ME): 50 mM HEPES-KOH (pH 8), 0.2 mM EDTA-Na_2_, 5 mM DTT, 2 mM NAD, 5 mM malate, 25 μM NADH, 0.1 mM acetyl Coenzyme A, 4 mM MnCl_2_; for NADP-dependent malic enzyme (NADP-ME): 100 mM Tris-HCl (pH 7), 10 mM MgCl_2_, 0.5 mM NADP, and 10 mM malate; for NADP-dependent isocitrate dehydrogenase (ICDH): 100 mM Tricine-KOH (pH 8), 0.25 mM NADP, 5 mM MgCl_2_, and 5 mM isocitrate. In the case of malate dehydrogenase (MDH) due to its high activity supernatants were diluted 30 times.

For GS, 50 μL of sample supernatants were incubated during 30 min at 30°C with 100 μL of reaction buffer [50 mM Tris-HCl (pH 7.6), 20 mM MgSO_4_, 80 mM sodium glutamate, 6 mM hydroxilamine, 4 mM Na_2_-EDTA, and 8 mM ATP] and the reaction stopped with 150 μL of acid ferric mixture [0.5 M TCA, 2 N HCl, 120 mM FeCl_3_]. Samples were centrifuged at 2,128 g for 5 min, and γ-glutamylmonohydroxamate (γ-GHM) colorimetrically quantified in the supernatants at 540 nm.

### Gel blots

Sodium dodecyl sulfate/polyacrylamide gel electrophoresis (SDS/PAGE) was performed with a 12% (w/v) acrylamide resolving gel and a 4.6% (w/v) stacking gel in a vertical electrophoresis cell (Mini-Protean III; Bio-Rad). Equal amounts of proteins were loaded in each well and separated at 150 V for 150 min. Proteins were then transferred into nitrocellulose membranes by wet electroblotting (Bio-Rad). Antibodies used were anti-GS (1:2,000) and anti-GDH (1:5,000) and goat anti-rabbit IgG-HRP as secondary antibody (1:20,000). Proteins were visualized using the Pierce ECL Western Blotting substrate (Thermo Scientific). Two bands were detected with anti-GS corresponding to GS1 and GS2. With anti-GDH we only detected a single band. The densitometry of the bands was calculated using the Image J software. The relative quantification was done respect to the most intense band of each blot (value “1”).

### RNA extraction and Q-RT-PCR analysis

Leaves and roots were homogenized in liquid nitrogen and total RNA was isolated using the Nucleospin RNA plant kit (Macherey-Nagel) according to the manufacturer's recommendations. RNA quality was checked and 1 μg of RNA retrotranscribed into cDNA using the PrimeScript™ RT reagent Kit (Takara Bio Inc.). Gene expression was measured by quantitative PCR in a 15 μL reaction using the SYBR Premix ExTaq™ Takara Bio Inc.) in a Step One Plus Real Time PCR System (Applied Biosystems) and 2 μL of cDNA diluted 1:10. The primers used for *gln* and *gdh* expression are described in Lothier et al. (2011) and Fontaine et al. (2012), respectively. The PCR program used was as follows: polymerase activation (95°C for 5 min), amplification and quantification cycles repeated 40 times (94°C for 15 s, 60°C for 1 min), and melting curve (40–95°C with one fluorescence read every 0.3°C). Relative expression was calculated as the ΔCp between each gene and the average of the housekeeping genes *SAND family* (At2g28390) and β*-tubulin 4* (At5g44340) with the primers described in Marino et al. (2013). Average ΔCp was calculated from three samples (each one representing a pool of three plantlets).

### Statistical analysis

Data were analyzed using SPSS 17.0 (Chicago, IL, USA). Statistical analysis of normality and homogeneity of variance were analyzed by Kolmogorov-Smirnov and Levene tests. Analysis of significant differences within each nitrogen dose included one-way ANOVA and comparison of means (Duncan's test). Nitrogen dose effect was carried out by t-student statistical analysis. Relationships between variables were tested by Pearson's correlation. Additional details about statistical analyses and significance levels are presented in figure legends.

## Results

*A. thaliana* Col-0 plants were grown for 12 days under ammonium nutrition in axenic hydroponic conditions to avoid the possibility of nitrification. Nitric nutrition was used as a reference for comparison. It should be noted that due to *A. thaliana 's* sensitivity to ammonium nutrition most of the studies published in relation to ammonium stress applied a mixed nutrition of nitrate with increasing concentrations of ammonium; however, as stated earlier, in this work ammonium was applied as the sole N-source.

**Figure 1:**
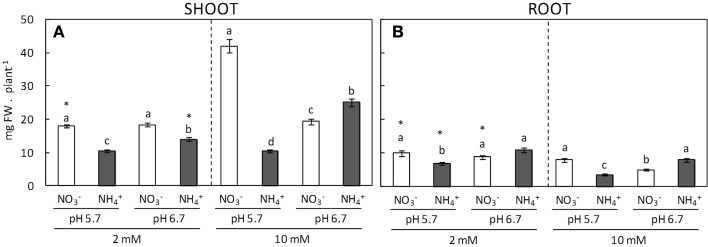
**Shoot (A) and root (B) biomass of plants grown under different conditions of pH (5.7 or 6.7), N source (${\text{NO}}_{3}^{-}$ or ${\text{NH}}_{4}^{+}$), and concentration (2 or 10 mM)**. Letters represent significant differences between treatments within the same N dose analyzed by Duncan's test (*p* < 0.05). Asterisk (^*^) represents the effect of N-dose between plants grown under the same pH and N source using a *t-student* test (*p* < 0.05). Columns represent mean ± se (*n* = 25–35).

Biomass production is surely the most comprehensive parameter used to evaluate plants performance in response to a long-term stressful situation. As expected, *A. thaliana* shoot biomass was overall reduced in ammonium-fed plants compared to equivalent nitrate-fed plants (Figure 1). This inhibition depended on the pH, since biomass accumulation was lower at pH 5.7, particularly at 10 mM dose. With respect to ${\text{NO}}_{3}^{-}$-fed plants, at 2 mM they grew at an equal rate in a pH independent manner whereas with 10 mM supply shoots biomass only presented a significant increase under pH 5.7 (Figure 1A). Root biomass and length responded to the different nutritional regimes in a similar manner as the shoots; however, these parameters were lesser at 10 mM according to the reduced need of surface exploration to acquire nutrients (Figure 1B; Supplementary Figure 2).

**Figure 2:**
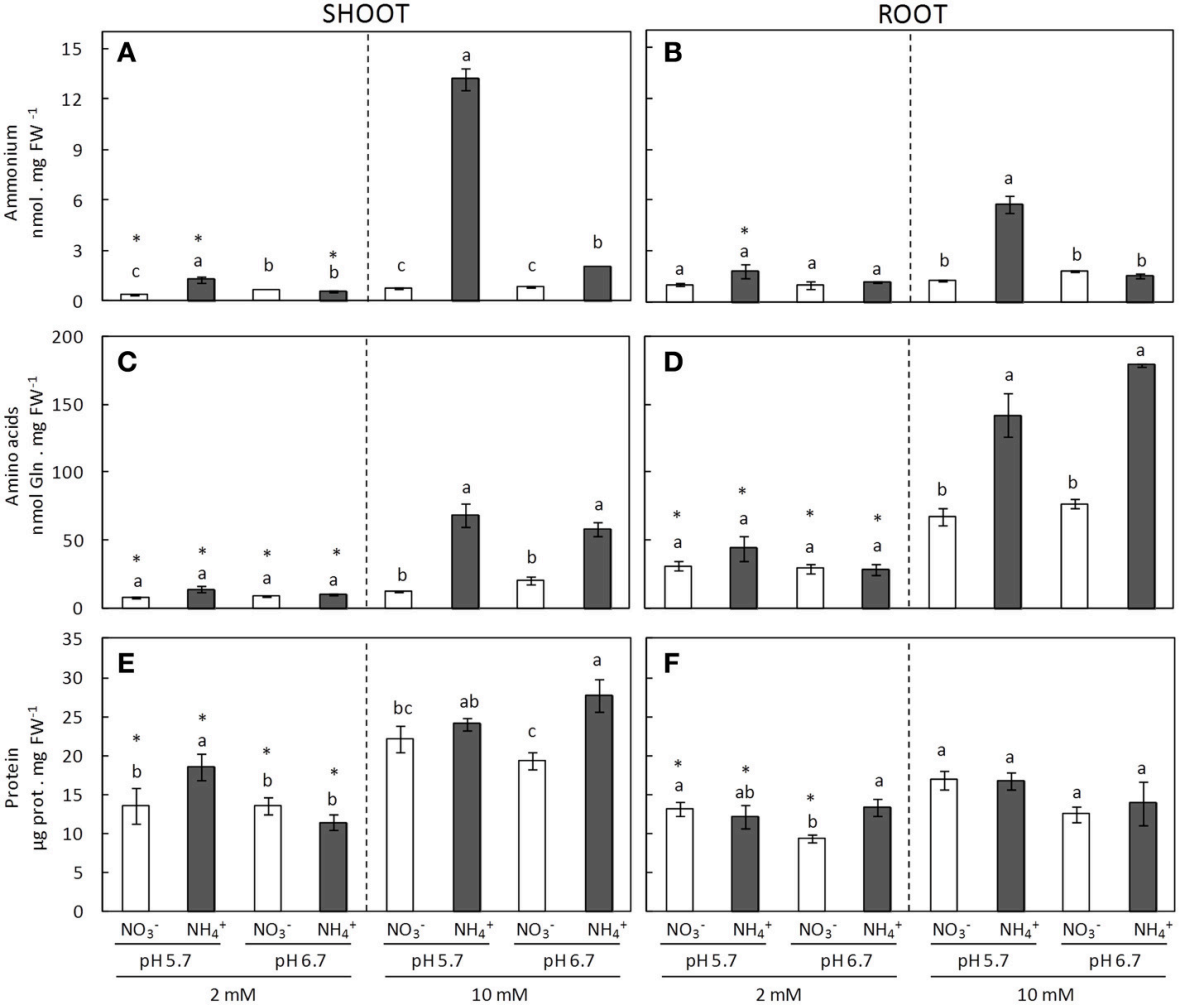
**Ammonium (A,B), amino acids (C,D), and protein content (E,F), of shoots (A,C,E) and roots (B,D,F) of plants grown under different conditions of pH (5.7 or 6.7), N source (${\text{NO}}_{3}^{-}$ or ${\text{NH}}_{4}^{+}$), and concentration (2 or 10 mM)**. Statistical analysis was described in Figure 1. Columns represent mean ± se (*n* = 3). Each sample is a pool of three plants.

${\text{NH}}_{4}^{+}$ content in both shoots and roots increased in ${\text{NH}}_{4}^{+}$-fed plants mainly under 10 mM dose. Interestingly, the degree of ammonium stress, estimated from the biomass, correlated to ${\text{NH}}_{4}^{+}$ accumulation; under pH 5.7 ammonium accumulation was around six and five times higher in shoots and roots, respectively, in comparison with plants grown at pH 6.7 (Figures 2A,B). An increase in the total free amino acid content is a typical response to ammonium nutrition (Britto and Kronzucker, 2002; Sarasketa et al., 2014). When the supplied nitrogen dose was 10 mM the increase in amino acid content under ammonium nutrition compared to nitrate nutrition was evident (Figures 2C,D). However, no differences were detected when comparing the effects of pH. Besides, amino acid content was always higher in roots compared to shoots (Figures 2C,D). Protein accumulation did not show any clear trends in function of the different nutritional conditions (Figures 2E,F); nevertheless, protein accumulation was notably greater in some ammonium treatments compared to nitrate counterparts, such as in shoots grown at 2 mM pH 5.7 and at 10 mM pH 6.7 (Figure 2E). Interestingly, the roots revealed a capacity to accumulate high levels of amino acids, while leaves preferentially accumulated ${\text{NH}}_{4}^{+}$ in the form of soluble proteins (Figure 2).

**Figure 3:**
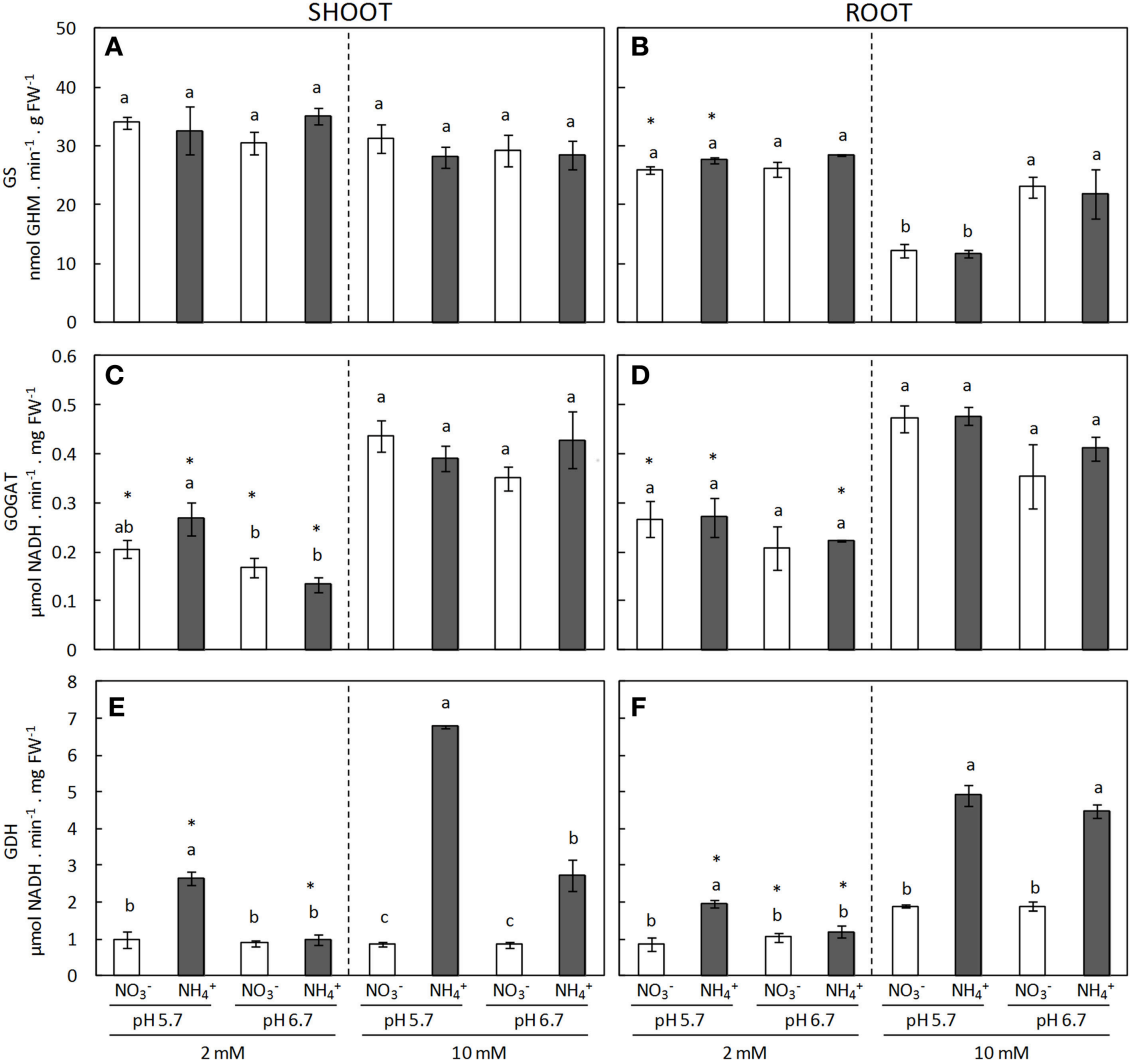
**GS (A,B), GOGAT (C,D), and GDH (E,F) enzyme activities from shoots (A,C,E) and roots (B,D,F) of plants grown under different conditions of pH (5.7 or 6.7), N source (${\text{NO}}_{3}^{-}$ or ${\text{NH}}_{4}^{+}$) and concentration (2 or 10 mM)**. Statistical analysis was described in Figure 1. Columns represent mean ± se (*n* = 3–6). Each sample is a pool of three plants.

The GS/GOGAT cycle is the main ammonium assimilation pathway. GS activity in shoots did not vary in response to the N source, concentration or pH (Figure 3A). Contrastingly, in roots at 10 mM dose, GS activity was greater under pH 6.7 compared to pH 5.7, regardless of the N source (Figure 3B). Control over the cycle has mainly been attributed to GS but a recent paper reported that the NADH-GOGAT enzyme in roots could be involved in ammonium tolerance (Konishi et al., 2014) and so we also included this enzyme in our study. NADH-GOGAT activity increased in both roots and shoots in response to N dose independent of the N source and pH (Figures 3C,D). On the other hand, GDH enzyme activity was clearly induced under ammonium nutrition compared with nitrate nutrition (Figures 3E,F). Overall, this induction was consistently more marked at pH 5.7 than pH 6.7. For instance, at 2 mM regime at pH 5.7 GDH activity in both shoots and roots of ammonium-fed plants was twice that of their nitrate counterpart (Figures 3E,F). Similarly, at 10 mM dose and pH 5.7 GDH activity in ${\text{NH}}_{4}^{+}$-fed shoots was nearly eight times higher than in those under nitrate nutrition, whereas at pH 6.7 the activity was only three times higher (Figure 3E). In roots, at high ${\text{NH}}_{4}^{+}$-dose GDH activity doubled that of those cultured with nitrate regardless of the external medium pH (Figure 3F). Interestingly, GDH activity in shoots was correlated with tissue ${\text{NH}}_{4}^{+}$ content highlighting the tight relationship between these two parameters (Supplementary Figure 3). GDH activity determined in its deaminating sense showed a similar trend as the one observed in its aminating sense (Supplementary Figure 4).

**Figure 4:**
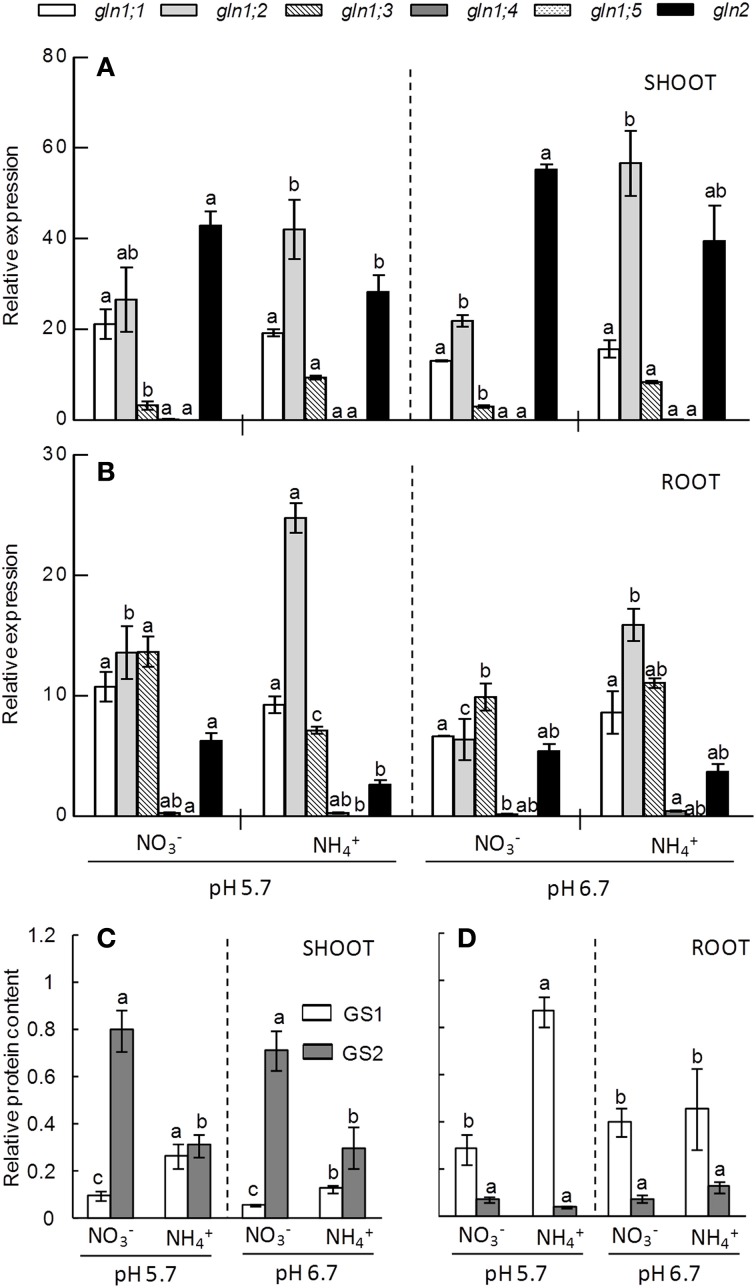
***GLN* genes expression pattern of shoots (A) and roots (B) and GS enzyme content of shoot (C) and root (D) of plants grown under 10 mM N concentration at pH 5.7 or 6.7 and ${\text{NO}}_{3}^{-}$ or ${\text{NH}}_{4}^{+}$ as N source**. Letters represent significant differences between treatments analyzed by Duncan's test (*p* < 0.05). Columns represent mean ± se (*n* = 3). Each sample is a pool of three plants. In Supplementary Figure 6 a zoom of *gln1;4* and *gln1;5* genes expression is available.

To further analyze how pH and N-source affected GS and GDH and how their activity relates to the different isoforms we determined their protein content and gene expression when grown under 10 mM nitrogen concentration. We chose this condition because at this N dose the effect of external medium pH on plants response under ammonium stress was more evident. In *A. thaliana* the cytosolic GS1 isoform is encoded by five genes (*gln1;1* to *gln1;5*). In shoots *gln1;1* and *gln1;2* were the genes that showed higher expression levels, while in roots *gln1;3* expression was also remarkable (Figures 4A,B). Ammonium nutrition provoked *gln1;2* induction in both shoots and roots under both pH regimes (Figure 4A). In addition, *gln1;3* was also induced by ammonium nutrition in shoots; however, in roots grown at pH 5.7 the expression was higher under nitrate nutrition (Figures 4A,B). According to *gln1* genes expression, GS1 protein content accumulated in both tissues when cultured under ammonium nutrition particularly when the external medium pH was pH 5.7 (Figures 4C,D). Nitrate nutrition induced the expression of plastidic GS2 in both shoots and roots (Figures 4A,B). However the content of GS2, as detected by western blotting, was only higher in the shoots of ${\text{NO}}_{3}^{-}$-fed plants (Figure 4). As expected, the most abundant GS isoform in shoots was GS2, while in roots it was GS1; however, due to the induction of *gln1* genes, GS1 and GS2 were present at similar levels in shoots of ammonium-fed plants at pH 5.7 (Figures 4C,D).

**Figure 5:**
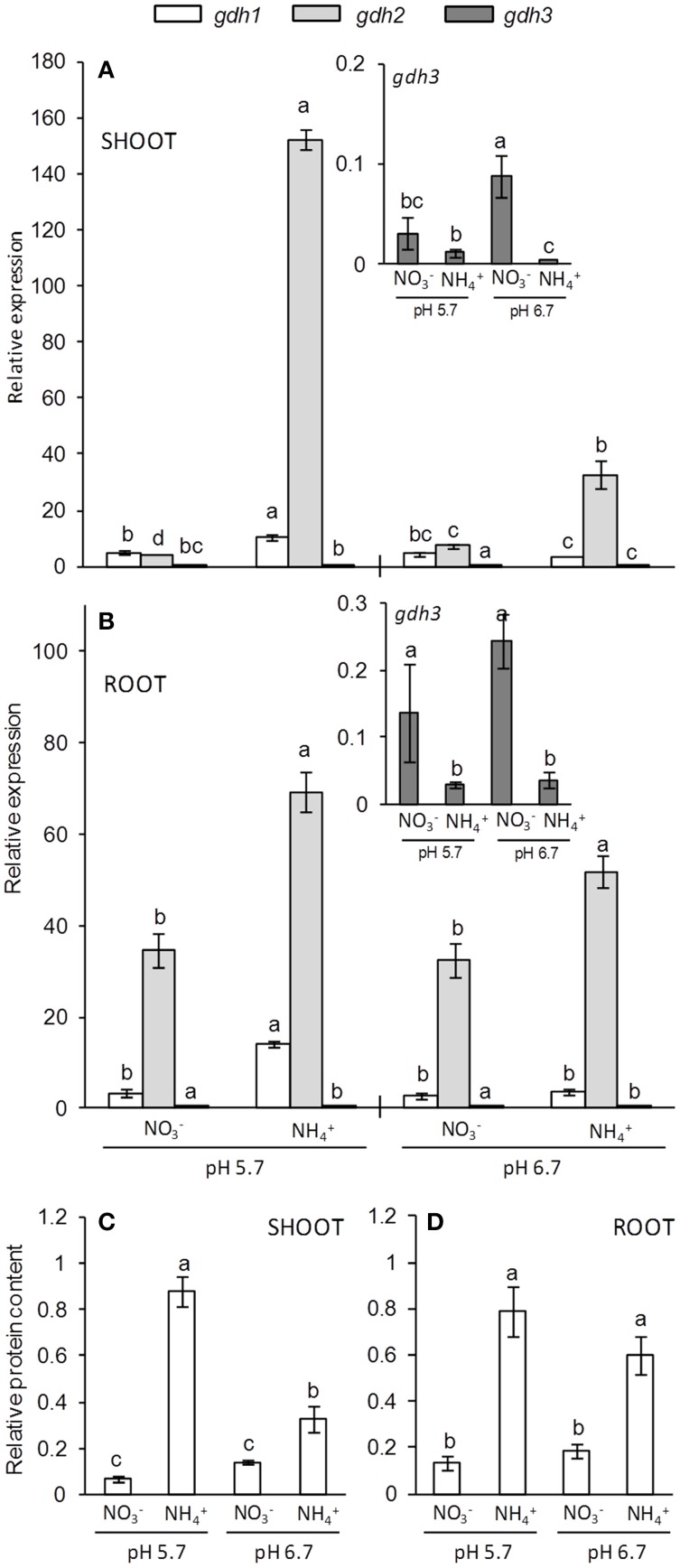
***GDH* genes expression (A,B) and protein content (C,D) of shoots (A,C) and roots (B,D) of plants grown under 10 mM N concentration at pH 5.7 or 6.7 and ${\text{NO}}_{3}^{-}$ or ${\text{NH}}_{4}^{+}$ as N source**. The insets show the details for *gdh3* expression. Letters represent significant differences between treatments analyzed by Duncan's test (*p* < 0.05). Columns represent mean ± se (*n* = 3). Each sample is a pool of three plants.

NAD(H)-GDH in Arabidopsis is encoded by three genes (*gdh1* to *gdh3*). A fourth gene encoding an NADP(H)-dependent GDH isoform has been described but this isoform seems to be inactive (Fontaine et al., 2012). In this work, *gdh2* was the most expressed gene in both shoots and roots while ammonium nutrition further induced its expression in both tissues (Figures 5A,B). Again, this induction was more pronounced at pH 5.7, the conditions under which biomass was more affected by ammonium stress. Moreover, *gdh1* expression was also induced in ammonium-fed plants but only at pH 5.7. Interestingly, the *gdh3* gene, whose expression was much lower than that of *gdh1* and *gdh2*, was induced in both shoots and roots under nitrate nutrition (Figures 5A,B). According to the increased expression of genes, GDH protein content was also greater under ammonium nutrition in both shoots and roots, with the highest induction observed in shoots at pH 5.7 (Figures 5C,D).

**Figure 6:**
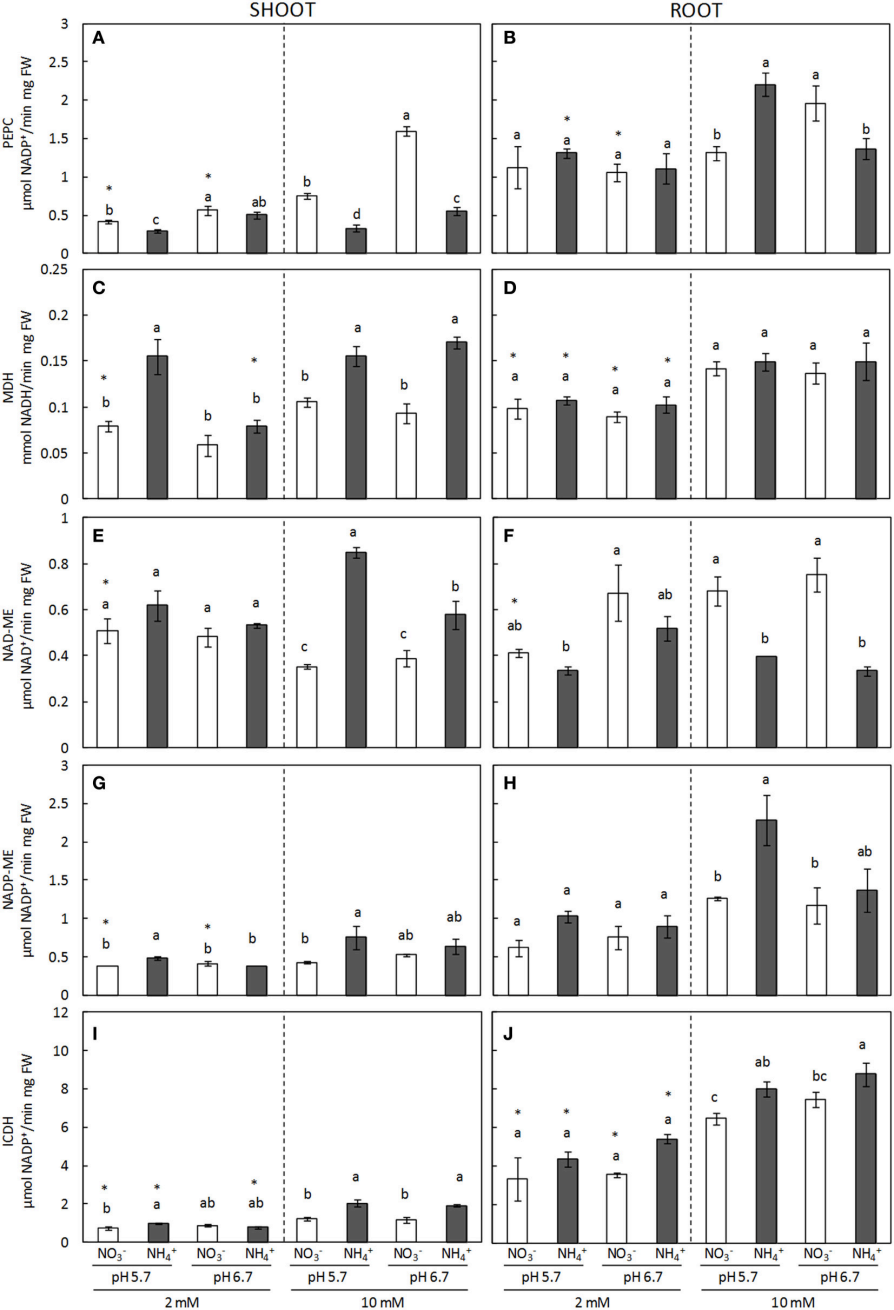
**PEPC (A,B), MDH (C,D), NAD-ME (E,F), and NADP-ME (G,H) and ICDH (I,J) enzyme activities from shoots (A,C,E,G,I) and roots (B,D,E,H,J) of plants grown under different conditions of pH (5.7 or 6.7), N source (${\text{NO}}_{3}^{-}$ or ${\text{NH}}_{4}^{+}$) and concentration (2 or 10 mM)**. Statistical analysis was described in Figure 1. Columns represent mean ± se (*n* = 3–6). Each sample is a pool of three plants.

TCA cycle anaplerotic enzymes presented a differential behavior depending on the organ. ICDH, MDH, NAD-ME, and NADP-ME activities were all induced in the shoots of plants grown under ammonium nutrition regardless of the external medium pH (Figure 6). This induction was generally greater under regimes which involved a higher degree of ammonium stress. For example, at 2 mM dose, ICDH and MDH induction was significant at pH 5.7, while at pH 6.7 it remained at the same level as that of nitrate-fed plants (Figures 6C,I). On the other hand, the effect of a higher ammonium concentration on the induction of TCA enzymes was evident; for example, it can be observed that NAD-ME activity remained stable at 2 mM dose while it was clearly induced under 10 mM ammonium dose (Figure 6E). Conversely, PEPC activity was greater in shoots of ${\text{NO}}_{3}^{-}$-fed plants, particularly when cultured at 10 mM concentration (Figure 6A). In roots, NADP-ME and ICDH activities responded in a similar manner as to the behavior observed in shoots, with the highest level of induction reported at 10 mM ${\text{NH}}_{4}^{+}$ and a pH of 5.7 (Figures 6B,F). Interestingly, the behavior of NAD-ME and PEPC changed significantly when comparing shoots against roots. NAD-ME activity was induced in shoots under ammonium nutrition, while in roots it was higher under nitrate nutrition (Figures 6E,F); and PEPC activity, which was greater in nitrate-fed shoots, was induced in ammonium-fed roots at 10 mM dose and pH 5.7 (Figures 6A,B).

## Discussion

The control of external medium pH has been shown to improve Arabidopsis tolerance to ammonium-induced stress (Britto and Kronzucker, 2002; Hachiya et al., 2012; Zheng et al., 2015). As expected, in our work we also found that the growth of ammonium-fed plants was improved when cultured at pH 6.7 compared to pH 5.7 (Figure 1). Indeed, the importance of pH regarding ammonium stress has also been highlighted by the use of Arabidopsis mutants with an altered ammonium tolerance. For instance, *vtc1*, mutant deficient in GDP-mannose pyrophosphorylase (Kempinski et al., 2011); *frostbite1*, mutant of mitochondrial respiratory chain Complex I (Podgorska et al., 2015); or *slah3*, mutant of the anion channel SLAC1 Homologue 3 (Zheng et al., 2015), all showed phenotypes under ammonium nutrition that were at least partially related to the control of external medium pH. Besides, Arabidopsis is very sensitive to ammonium nutrition and ammonium stress has commonly been induced by applying increasing concentrations of ammonium concomitantly with nitrate usually in proportions of between 4:1 and 12:1 (ammonium:nitrate). The reasons behind the nitrate-dependent alleviation of ammonium stress are not yet fully understood, but it has been suggested that it could be related to pH regulation (Hachiya et al., 2012). In addition, plasma membrane H^+^-ATPases activity is closely related to ion uptake compensating charge movements and the energy needed to feed H^+^-ATPases has been associated with poor root growth in a species-dependent manner at acidic pH values of around 3.5 (Yan et al., 1992, 1998). Indeed, one reported response of ammonium nutrition is to increase H^+^-ATPase activity (Yamashita et al., 1995; Zhu et al., 2009) and thus the energy consumed to maintain H^+^-ATPase could be involved in the higher stress degree commonly observed at acidic pHs. Therefore, all these data underline the importance of studying the relation between external medium pH and ammonium nutrition. In the present study, we focused, mainly by examining ${\text{NH}}_{4}^{+}$ assimilation and TCA cycle anaplerotic enzymes, on how the metabolism of Arabidopsis plants, adapts to different degrees of ammonium stress.

At pH 6.7 the ammonium stress was alleviated and so at this pH Arabidopsis plants responded positively to an increase in external ammonium concentration whereas at pH 5.7 plants yielded a reduced biomass (Figure 1). At pH 6.7 nitrate-fed plants did not respond to an increase in N-dose and were therefore significantly smaller than those grown at the same concentration of nitrate but at pH 5.7 (Figure 1). Previous studies have also observed impaired growth of nitrate-fed plants in response to medium alkalinization across similar pH ranges, for example, in maize (Schortemeyer et al., 1993), *Typha latifolia* (Brix et al., 2002), or tomato (Zhao and Ling, 2007). Thus, it seems that the availability of essential nutrients could be responsible for this pH-dependent growth effect in plants fed with 10 mM nitrate. In our study, we did not observe any significant alterations in the metabolic parameters analyzed that could explain such growth differences (Figures 2–5). Future work will help to elucidate the negative effect that certain plant species experience in relation to nitrate nutrition and external medium alkalinization.

In several works, acidic pHs have been shown to induce ammonium uptake or accumulation in tissues (Chaillou et al., 1991; Søgaard et al., 2009; Ortiz-Ramirez et al., 2011; Coskun et al., 2013). In our study, the degree of ammonium stress was correlated with ${\text{NH}}_{4}^{+}$ tissue accumulation since both roots and shoots accumulated much more ammonium at pH 5.7 compared to pH 6.7 (Figure 2). And so ${\text{NH}}_{4}^{+}$ accumulation could be due to ammonium transport rather than a result of impairing the metabolic pathways involved in its assimilation, as the contents of both amino acids and proteins were at similar levels in ammonium-fed plants regardless of the external medium pH (Figure 2).

It is known that ammonium assimilation is mainly driven by the GS/GOGAT cycle. Concerning GDH, there is still controversy about its role in plants but it is now accepted that GDH activity *in vivo* is primarily directed toward 2-oxoglutarate production (Labboun et al., 2009; Fontaine et al., 2012). However, under some circumstances it seems that GDH might also be collaborating in the direct amination of 2-OG to form glutamate, such as during fruit ripening (Ferraro et al., 2015) or ammonium stress (Skopelitis et al., 2006). It is apparent that an increased capacity to assimilate ammonium would help to prevent ${\text{NH}}_{4}^{+}$ content rising to toxic levels while simultaneously increasing plant growth potential. Indeed, GS1 overexpression in tobacco plants accumulated less ${\text{NH}}_{4}^{+}$ than wild-type plants under nitrate-based nutrition (Oliveira et al., 2002). Similarly, it has been proposed that plants which are capable of maintaining high levels of GS activity in the dark present an enhanced tolerance to ammonium stress (Cruz et al., 2006). In the present work, neither GS nor NADH-GOGAT activities presented any response to a different N-source. Contrastingly, GDH clearly showed an overall induction under ammonium nutrition. This induction was greatest in shoots at pH 5.7, where ammonium accumulation was higher; suggesting that GDH induction in the shoot depends on stress severity (Figure 3).

Different functions have been proposed for different GS and GDH isoforms (Lothier et al., 2011; Marchi et al., 2013; Guan et al., 2015). The main function of GS2 has been associated to the reassimilation of photorespiratory ammonium in photosynthetic tissues (Pérez-Delgado et al., 2015) and primary nitrogen assimilation in green tissues (Xu et al., 2012). Considering that ${\text{NO}}_{2}^{-}$ is reduced to ${\text{NH}}_{4}^{+}$ in the chloroplasts, we expected to encounter higher GS2 levels in nitrate-fed plants compared to ammonium-fed plants (Figure 4) as this has previously been observed in other plants including Arabidopsis (Sarasketa et al., 2014) or maize (Prinsi and Espen, 2015). GS1 content was only higher under the more toxic ammonium treatment in relation with increased *gln1;2* and *gln1;3* gene expression. Interestingly, Arabidopsis *gln1;2* mutants grown *in vitro* were about 20% smaller than wild-type plants grown under ammonium nutrition (Lothier et al., 2011). Similarly, rice mutants lacking OsGS1:1 experienced growth retardation under ammonium nutrition (Kusano et al., 2011). Overall, GS1 is essential under ammonium nutrition and different isoforms present non-overlapping functions. However, GS activity is subjected to tight post-transcriptional and post-translational regulation by, among others, phosphorylation (Prinsi and Espen, 2015) or nitration (Melo et al., 2011); and these regulatory mechanisms could explain the observation that GS activity did not vary in function of the N-source (Figure 2), contrary to its genes expression levels or protein content (Figure 4).

Ammonium has been known to induce GDH activity for decades, while heavier hexamers (enriched in α subunits) are often induced by ammonium (Cammaerts and Jacobs, 1985; Skopelitis et al., 2006). In our work, *gdh1* and *gdh2* were induced in response to ammonium nutrition, but interestingly *gdh1* was only induced at pH 5.7, and *gdh2* induction in shoots was greater at pH 5.7 than pH 6.7. This suggests that the observed increase in GDH protein content and activity was due to the induction of both genes (Figures 3, 5). Interestingly, expression of the until recently unstudied *gdh3* gene was higher in nitrate-fed plants, thus revealing a differential behavior for this isoform. Whether GDH3 could be playing a specific role under nitrate nutrition is still unknown. However, Marchi et al. (2013) proposed a role for GDH3 in nutrient remobilization during the Arabidopsis reproductive phase; furthermore, they showed *gdh3* induction by cytokinins, hormones known to regulate plant growth in response to nitrate (Krouk et al., 2011). Thus, our data suggest specific functions for the different GDH isoforms depending on both the type of N source and the degree of ammonium stress. Future research is still required to decipher the importance of GDH with regards to ammonium nutrition and to reveal the functional specificity of each isoform in plant metabolism. Overall, GS1, *gdh1* (encoding GDHβ) and *gdh2* (encoding GDHα) seem to be responding to the level of ammonium stress, which occurs to a higher extent at pH 5.7, and collectively suggest an important role of increased nitrogen assimilation capacity during ammonium nutrition. However, the content of total protein and amino acids did not accumulate at pH 5.7 compared to pH 6.7 suggesting that induction of N assimilation enzymes was not sufficient to scavenge the excess of ammonium into biomolecules. On the other hand, ammonium-fed plants may suffer from carbon limitation for ${\text{NH}}_{4}^{+}$ assimilation (Ariz et al., 2011; Setién et al., 2013; Vega-Mas et al., 2015) and it has been shown that the main function of GDH activity is to provide 2-oxoglutarate when C becomes limiting (Fontaine et al., 2012). In the present work, when correlating GDH activity with ${\text{NH}}_{4}^{+}$ accumulation (Supplementary Figure 3), we found a negative correlation in shoots for nitrate nutrition (*r* = −0.994, *p* = 0.006), while under ammonium nutrition this correlation was positive (*r* = 0.969, *p* = 0.031), which could be a sign that the role of GDH induction is directed toward 2-OG production to meet GS/GOGAT demand. However, the induction of GDH in response to ${\text{NH}}_{4}^{+}$ accumulation to collaborate in its assimilation cannot be discarded and future work using isotopic labeling together with mutant analysis under ammonium stress will surely help to shed more light on GDH function.

TCA cycle anaplerotic enzymes induction has been revealed important in order to counteract the depletion of TCA intermediates diverted to ${\text{NH}}_{4}^{+}$ assimilation; thus, they are crucial upon ammonium nutrition. Indeed, organic acids and malate pools decline in correlation with an increase in amino acid content has often been observed under ammonium nutrition (Britto and Kronzucker, 2005; Setién et al., 2013). In the present work, MDH, NAD-ME, and NADP-ME were induced in shoots and could play a role in organic acids consumption (Figure 5). Furthermore, shoot NAD-ME and root NADP-ME induction was greater under a harsher degree of ammonium stress. Interestingly, NAD-ME levels in the roots were induced by nitrate nutrition and the plastidic and mitochondrial localization of this enzyme (Maier et al., 2011) may suggest a differential localization or function of malate pool in function of the N source. ICDH is a key enzyme in the provision of 2-OG, in the present study it was also induced in response to ammonium nutrition (Figure 5), as it has been observed in other plants such as pea (Ariz et al., 2013). In line with ICDH's key role in 2-OG production, the amino acids content in shoots was observed to positively correlate with ICDH activity (Supplementary Figure 5). The importance of this enzyme was evident in plants lacking total or partial ICDH expression, since they presented reduced pools of 2-OG under carbon limitation (Boex-Fontvieille et al., 2013). On the other hand, ammonium nutrition is known to provoke redox alterations (Podgorska et al., 2013) and ICDH function supplying NADPH has also been related to redox homeostasis control (Marino et al., 2007; Mhamdi et al., 2010), thus the possibility that ICDH induction could also be related to cell redox control cannot be ruled out. With regards to PEPC, it has recently been shown that ammonium assimilation was impaired in the Arabidopsis *PEPC* double mutant *ppc1/ppc2* grown in standard 1/2 MS medium (Shi et al., 2015) and, although disparate results have been found in different species, PEPC is known to be induced under ammonium stress, mainly in roots (Lasa et al., 2002; Britto and Kronzucker, 2005; Ariz et al., 2013). In the present study, higher PEPC activity in shoots of nitrate fed-plants will corroborate the need to replenish carbon intermediates in shoots when nitrate is the N source, whereas under ammonium stress, ${\text{NH}}_{4}^{+}$ assimilation would preferentially occur in the roots. Thus, fine regulation of TCA anaplerotic enzymes appears to be a key aspect when trying to improve plants ${\text{NH}}_{4}^{+}$ assimilation capacity under ammonium stress.

### Final conclusions

Variations in the pH of the external medium are known to affect plants N nutrition. Regarding ammonium nutrition, pH control appears to play a key role in determining plant ammonium tolerance or sensitivity. In Arabidopsis, external medium buffering or medium alkalinization has been shown to mitigate some of the detrimental effects associated with ammonium stress, but how plant cell metabolism adapts to those changes has barely been studied, especially in the roots. In the present work, the higher degree of ammonium stress was related to ${\text{NH}}_{4}^{+}$ accumulation at pH 5.7 which could not be circumvented by the induction of ammonium assimilation machinery, including TCA cycle anaplerotic enzymes. Moreover, this study suggests specific roles for different GS and GDH isoforms in function of the nutritional regime. Similarly, anaplerotic enzymes seem to play an important role at the interface between carbon and nitrogen metabolism and future studies into ammonium nutrition with the use of knockout mutants in the different TCA cycle anaplerotic enzymes will be extremely helpful in gaining a better understanding of their role in ammonium stress. Finally, fluxomic analysis, paying special attention to metabolites subcellular localization, will elucidate the changes occurring in plant cell metabolism under ammonium-based nutrition.

## Acknowledgments

AS is holder of a Ph.D. grant from the Basque Government. This research was financially supported by the Basque Government (IT932-16), the University of the Basque Country (EHUA14/14), the Spanish Ministry of Economy and Competitiveness (BIO2014-56271-R co-funded by FEDER) and the People Programme (Marie Curie Actions) of the European Union's Seventh Framework Programme (FP7/2007–2013) under REA grant agreement number 334019. We are also grateful to Dr. J. F. Moran and Dr. K. A. Roubelakis-Angelakis for lending GS and GDH antibodies, respectively.

## Author contributions

AS and DM performed experiments; AS, DM, MBG, and CG analyzed data, DM designed the experiment and wrote the paper.

### Conflict of interest statement

The authors declare that the research was conducted in the absence of any commercial or financial relationships that could be construed as a potential conflict of interest.

## Supplementary material

Supplementary Figure 1 **External medium pH monitoring during Arabidopsis plants growth under different conditions of pH (5.7 or 6.7), N source (${\text{NO}}_{3}^{-}$ or ${\text{NH}}_{4}^{+}$) and concentration (2 or 10 mM)**.

Supplementary Figure 2 **Root length of plants grown under different conditions of pH (5.7 or 6.7), N source (${\text{NO}}_{3}^{-}$ or ${\text{NH}}_{4}^{+}$), and concentration (2 or 10 mM)**. Statistical analysis was described in Figure 1. Columns represent mean ± se (*n* = 25–35).

Supplementary Figure 3 **Pearson correlations between GDH activity and tissue ${\text{NH}}_{4}^{+}$ content in roots and leaves of plants grown under nitrate or ammonium as nitrogen source**. Correlation lines are presented only if *p* < 0.05.

Supplementary Figure 4 **GDH enzyme activity measured on its deaminating sense from shoots (A) and roots (B) of plants grown under different conditions of pH (5.7 or 6.7), N source (${\text{NO}}_{3}^{-}$ or ${\text{NH}}_{4}^{+}$), and concentration (2 or 10 mM)**. Statistical analysis was described in Figure 1. Columns represent mean ± se (*n* = 3). Each sample is a pool of three plants.

Supplementary Figure 5 **Pearson correlations between ICDH activity and amino acid content in roots and leaves of plants grown under nitrate or ammonium as nitrogen source**. Correlation lines are presented only if *p* < 0.05.

Supplementary Figure 6 **Zoom of *gln1-4* and *gn1-5* genes expression shown in Figure 4**.
