# Supplementary figures and images for: Nitrogen Source and External Medium pH Interaction Differentially Affects Root and Shoot Metabolism in Arabidopsis
Source: Front Plant Sci. 2016 Feb 1;7:29. doi: 10.3389/fpls.2016.00029 (PMC4734181; doi:10.3389/fpls.2016.00029)

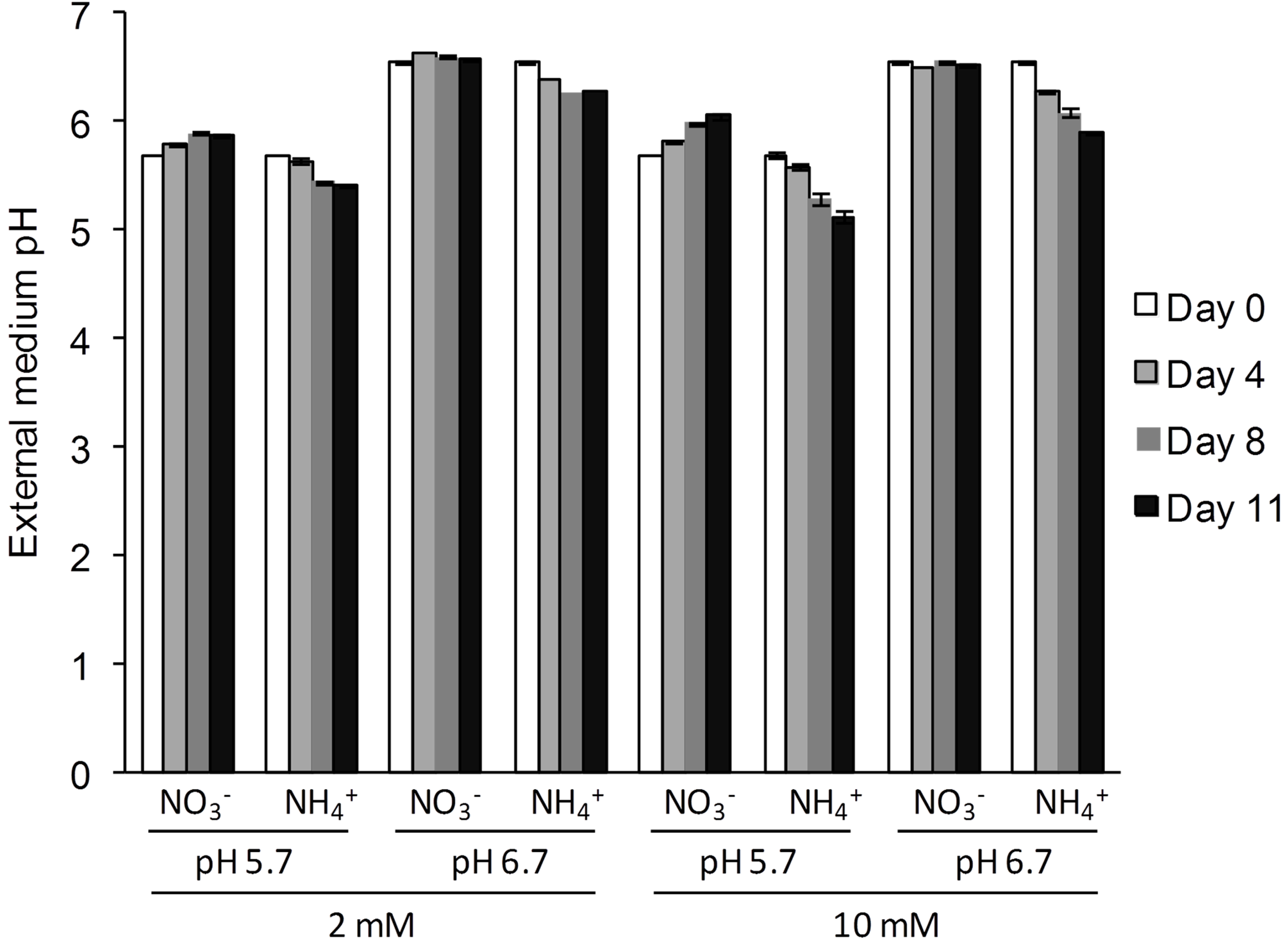

Supplement: Supplementary Figure 1 — External medium pH monitoring during Arabidopsis plants growth under different conditions of pH (5.7 or 6.7), N source (NO3- or NH4+) and concentration (2 or 10 mM). [file Image1.TIFF]

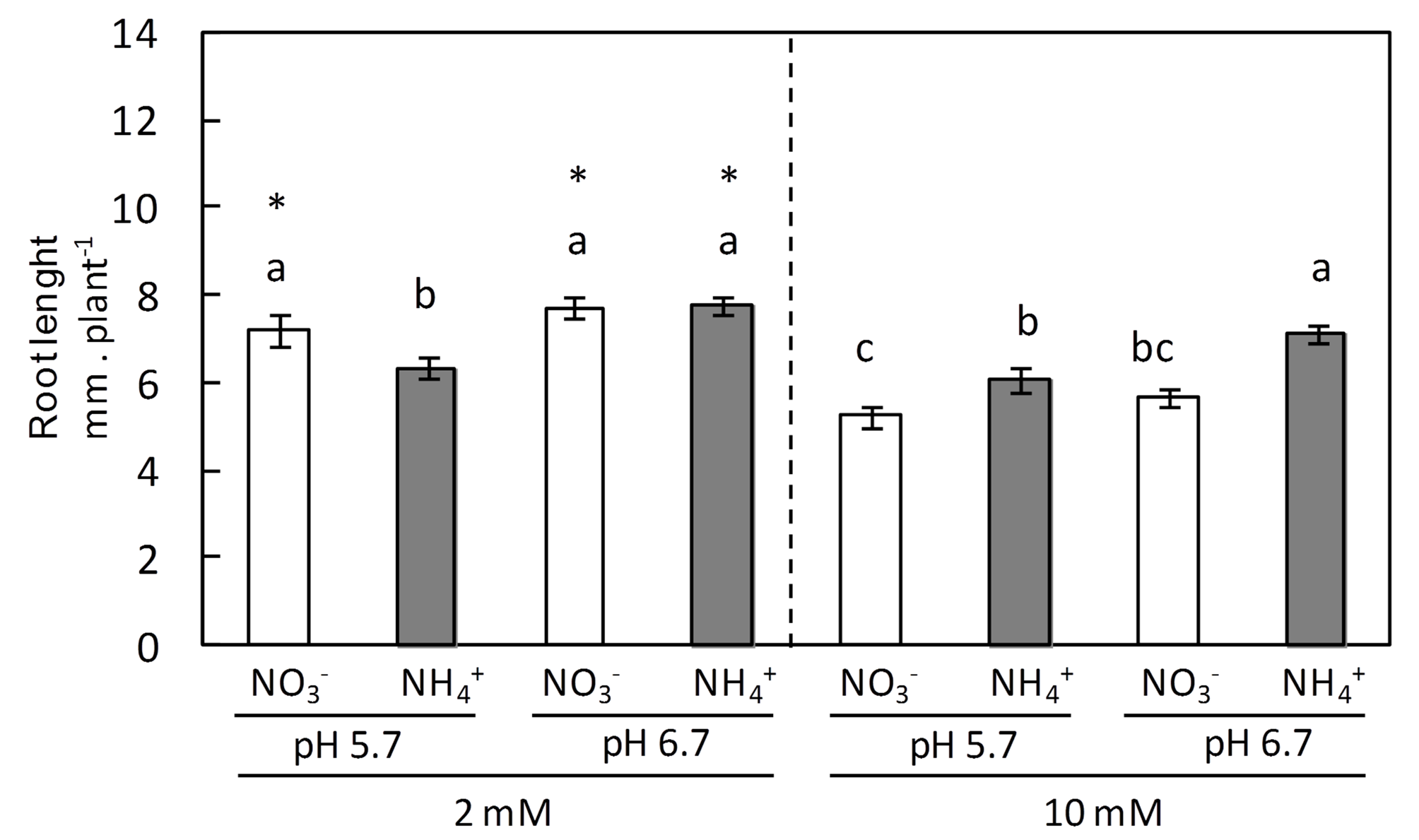

Supplement: Supplementary Figure 2 — Root length of plants grown under different conditions of pH (5.7 or 6.7), N source (NO3- or NH4+), and concentration (2 or 10 mM). Statistical analysis was described in Figure 1. Columns represent mean ± se (n = 25–35). [file Image2.TIF]

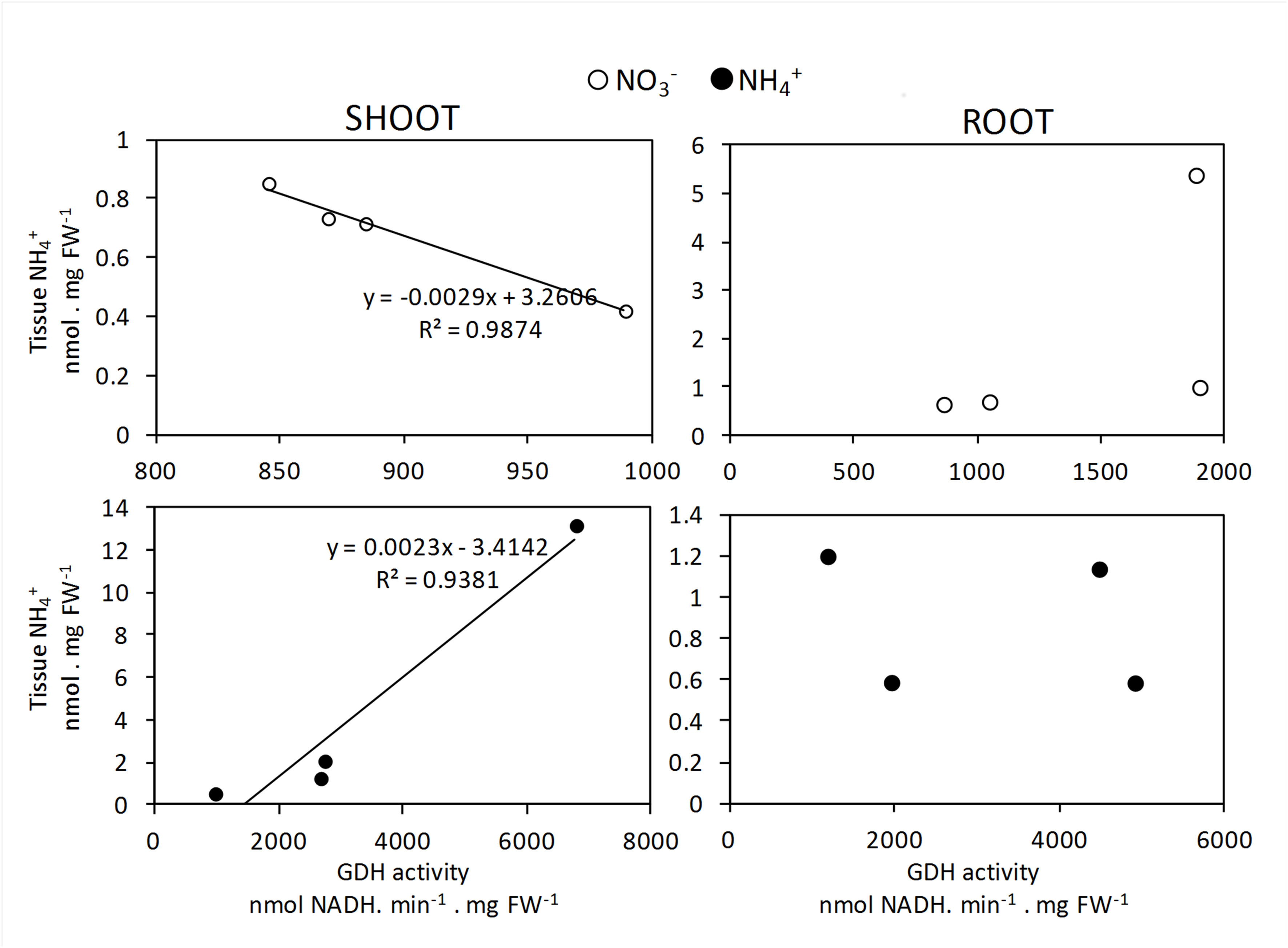

Supplement: Supplementary Figure 3 — Pearson correlations between GDH activity and tissue NH4+ content in roots and leaves of plants grown under nitrate or ammonium as nitrogen source. Correlation lines are presented only if p < 0.05. [file Image3.TIFF]

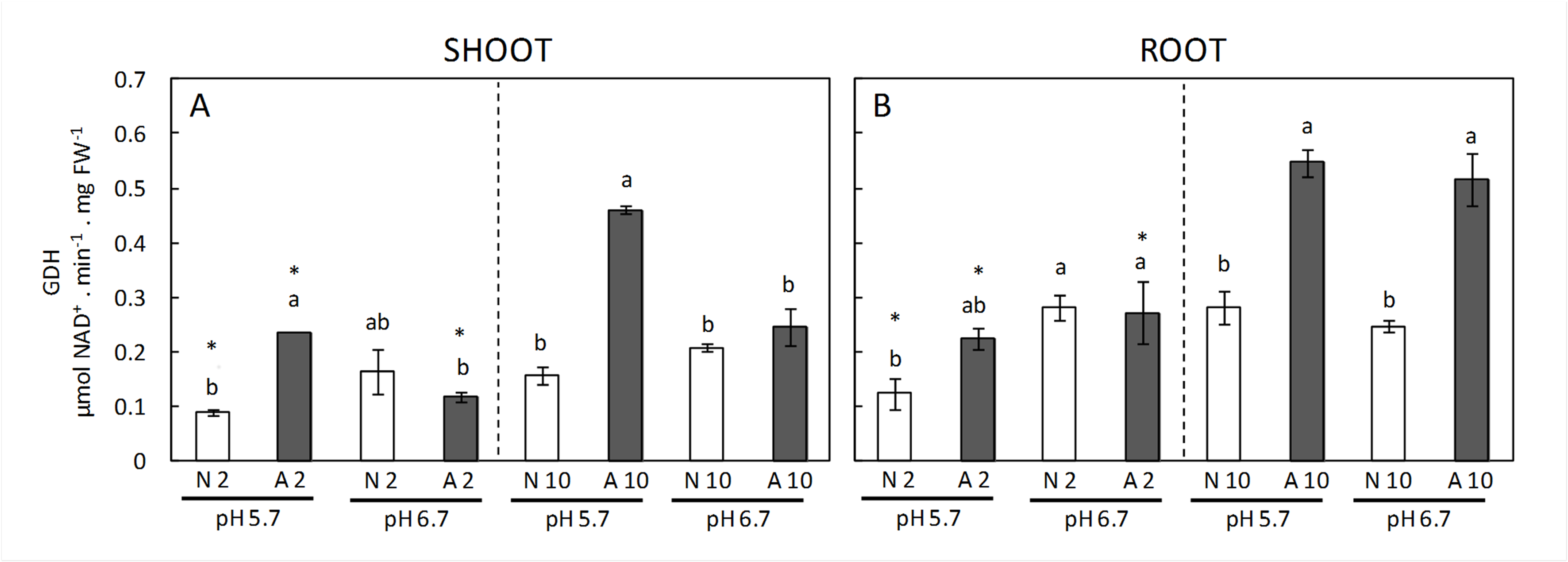

Supplement: Supplementary Figure 4 — GDH enzyme activity measured on its deaminating sense from shoots (A) and roots (B) of plants grown under different conditions of pH (5.7 or 6.7), N source (NO3- or NH4+), and concentration (2 or 10 mM). Statistical analysis was described in Figure 1. Columns represent mean ± se (n = 3). Each sample is a pool of three plants. [file Image4.TIFF]

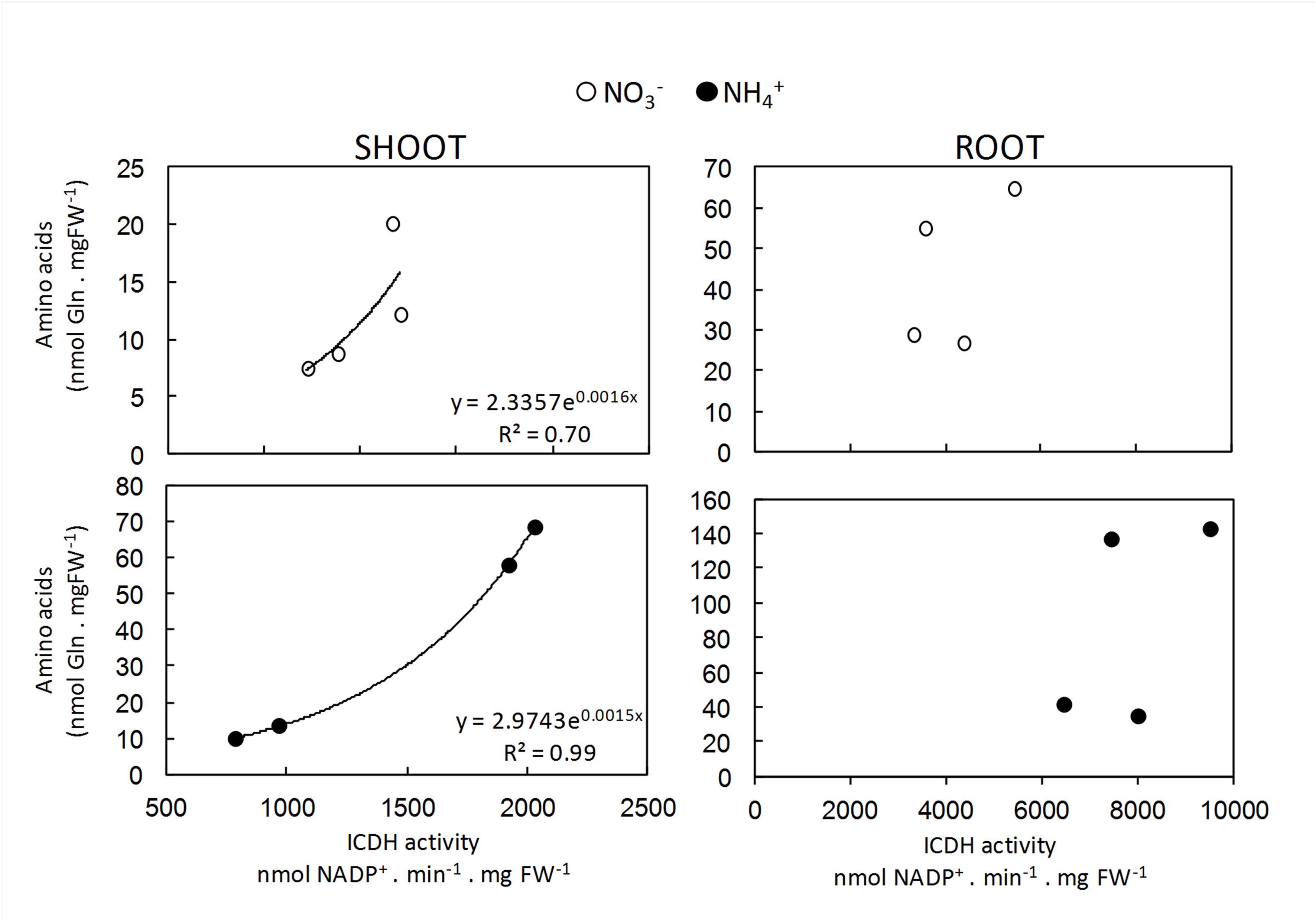

Supplement: Supplementary Figure 5 — Pearson correlations between ICDH activity and amino acid content in roots and leaves of plants grown under nitrate or ammonium as nitrogen source. Correlation lines are presented only if p < 0.05. [file Image5.TIFF]

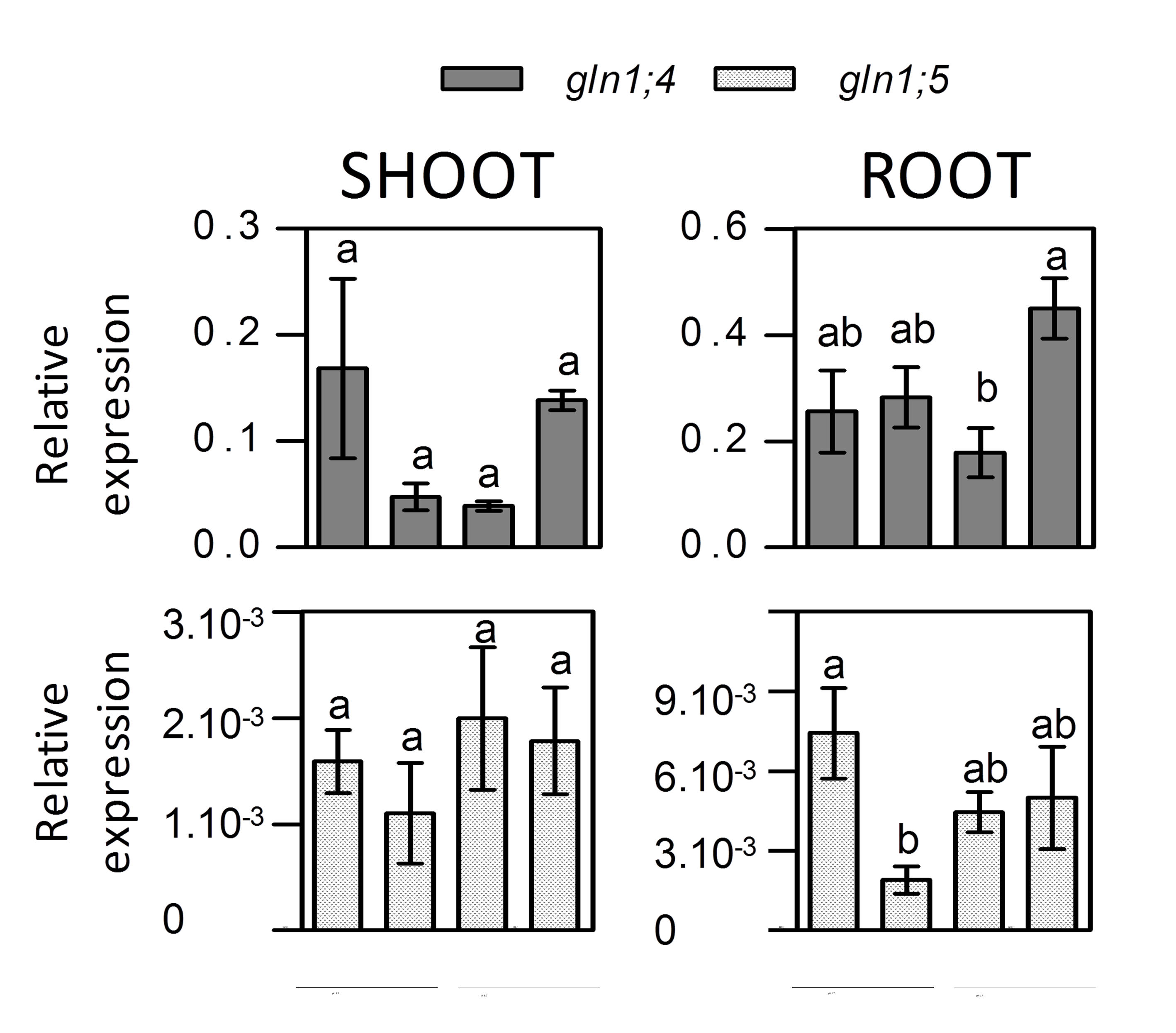

Supplement: Supplementary Figure 6 — Zoom of gln1-4 and gn1-5 genes expression shown in Figure 4. [file Image6.TIF]
